# Angioplasty for the treatment of left brachiocephalic vein aneurysm: a case report

**DOI:** 10.1186/s13019-021-01440-y

**Published:** 2021-03-31

**Authors:** Long Gui, Hongbo Xu, Chengdong Ning, Niuliu Huang, Yu Pan

**Affiliations:** Cardiothoracic Surgery Department, Lu’an People’s Hospital affiliated to Anhui Medical University, Lu’an City, Anhui Province China

**Keywords:** Brachiocephalic vein aneurysm, Innominate vein aneurysm, Angioplasty

## Abstract

**Background:**

Brachiocephalic vein aneurysm is a rare vascular malformation, which is often reported in case reports. At present it has attracted much attention due to the serious complications, such as vein aneurysm rupture, pulmonary embolism, venous thrombosis, etc. We report a case of left brachiocephalic vein aneurysm with compression symptoms.

**Case presentation:**

a 52-year-old male who was admitted to our hospital with irritating cough for more than 1 month. Chest contrast-enhanced CT showed a localized expansion of 5.2 cm in diameter of the left brachiocephalic vein in the anterior mediastinum. The patient received venous angioplasty with brachiocephalic vein aneurysm resection, and the postoperative recovery was well.

**Conclusion:**

Surgical operation is an effecive treatment method for brachiocephalic vein aneurysm, but it is still necessary to choose the appropriate way according to the type, size, location, lesion scope and complications of the vein aneurysm.

## Background

Brachiocephalic vein aneurysm (also known as innominate vein aneurysm) is a relatively rare vascular malformation, which mostly appears in the literature as case reports [1]. The exact etiology of brachiocephalic vein aneurysm remains undetermined. Possible etiologies include congenital developmental defects, malformations, trauma, inflammation, and degenerative changes of the vascular wall [2, 3]. It can be divided into primary and secondary. Primary venous aneurysms are due to the uneven development of vascular structure and defects in the adventitia muscle layer, which lead to the localized weakness and bulging of the vascular wall, in addition to cystic hygroma-related cases. The secondary is mainly due to excessive pressure in the brachiocephalic vein, excessive filling, or heart disease accompanied by blood return disorder. Clinically, it is divided into two subtypes, fusiform and cystic, according to its morphology. The former shows fusiform expansion in the brachiocephalic vein, and the latter shows cystic expansion, connected with the brachiocephalic vein with the aneurysm neck [4].

Brachiocephalic venous aneurysms often have no obvious clinical symptoms and signs, and are usually found due to abnormal imaging findings during physical examination. Some patients gradually develop compression symptoms due to the increase in aneurysm volume, such as post-activity chest tightness, wheezing, coughing after and pain behind the breastbone, etc. There are also a very small number of patients who seek medical treatment due to related complications (such as aneurysm rupture, pulmonary embolism, venous thrombosis, etc.). The incidence of complications and mortality increases with the increase of aneurysm cavity [5].

Here we report a case of surgical treatment due to brachiocephalic vein aneurysm.

## Case presentation

**Fig. 1:**
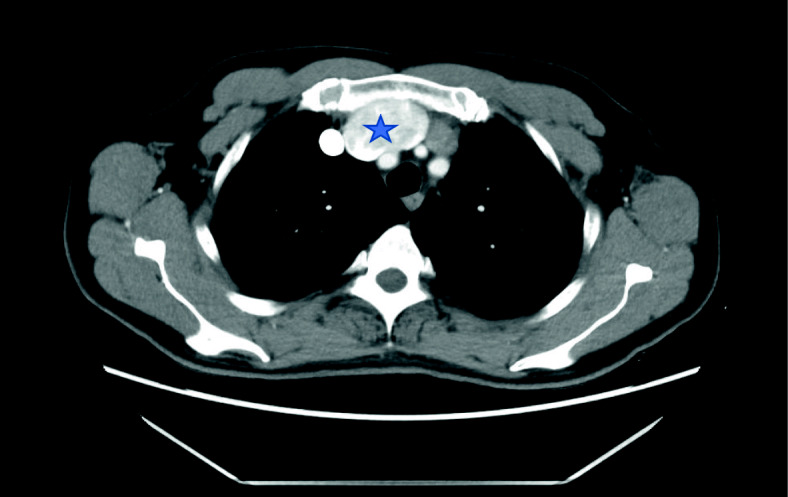
Chest-enhanced CT scan showed the location of the vein aneurysm, marked with a pentacle

A 52-year-old male patient was admitted to the hospital due to an irritating cough for more than 1 month. He had no recent history of chest trauma surgery or respiratory diseases. A chest CT scan showed anterior mediastinal space, and showed differential diagnosis of teratoma, thymoma and lymphomas, in order to clarify the relationship between the space-occupying lesions and blood vessels and surrounding tissues, a chest-enhanced CT examination was performed. After intravenous injection of contrast medium, a dual-phase scan was performed. It was found that there was a localized aneurysm-like expansion in the left brachiocephalic vein and its diameter was 5.2 cm with the smooth edge (Fig. 1), which confirmed the diagnosis of venous aneurysm. After careful evaluation of the patient, surgery was performed.

**Fig. 2:**
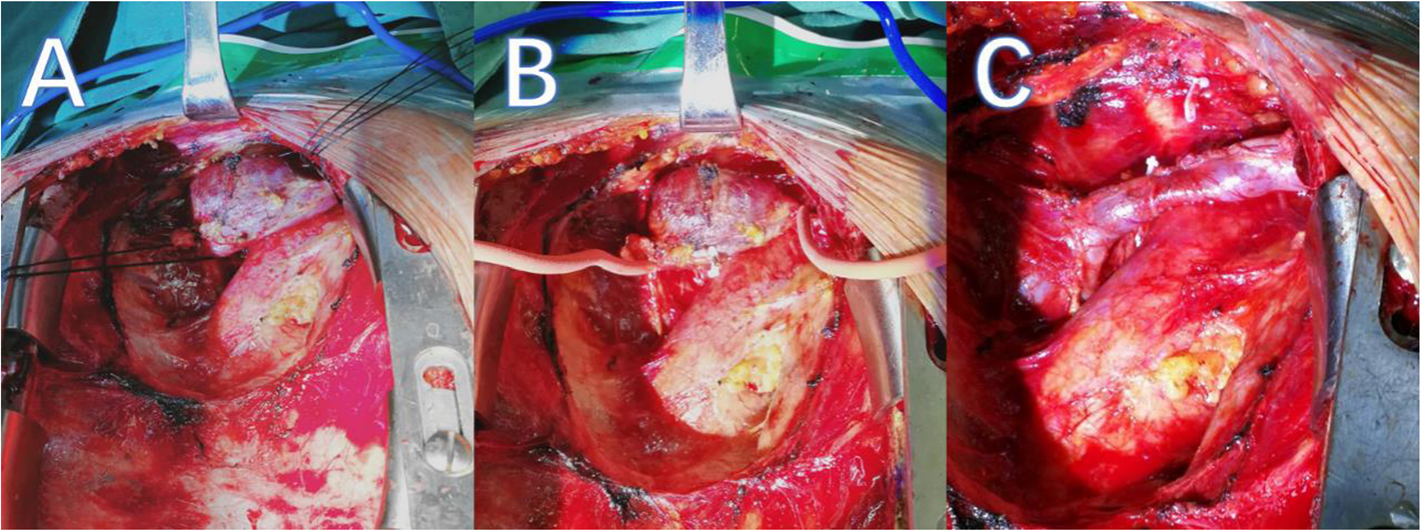
Intra-operative images of left brachiocephalic vein aneurysm. **a**: The aneurysm and surrounding tissues were carefully separated. **b**: Block both ends of the brachiocephalic vein. **c**: The left brachiocephalic vein aneurysm after angioplasty

A median sternum incision was selected in the surgery. During the operation, a huge aneurysm is found to be located in front of the left brachiocephalic vein. The aneurysm and surrounding tissues were carefully separated to prevent uncontrollable bleeding during the process, and to mechanical damage caused by extracorporeal circulation. We first performed heparinization of the whole body blood, and blocked both ends of the brachiocephalic vein, separated the fibrous membrane of the venous blood vessel, and found that the aneurysm wall was thin. The aneurysm was cystic expansion, and there as a larger aneurysm neck connected to it. After the aneurysm was removed, a double-layer continuous suture with 5–0 prolene thread was used to suture the brachiocephalic vein. We paid attention to the shape of the normal brachiocephalic vein during the suture process. To prevent vascular distortion and lumen stenosis, we carefully loosened the blocking bands at both ends before knotting, determined the shape of the formed blood vessel, and expelled the possible air (Fig. 2). After confirming that there is no obvious active bleeding, heparin was neutralized with protamine, and the operation ended after the sternum is fixed by the wire.

**Fig. 3:**
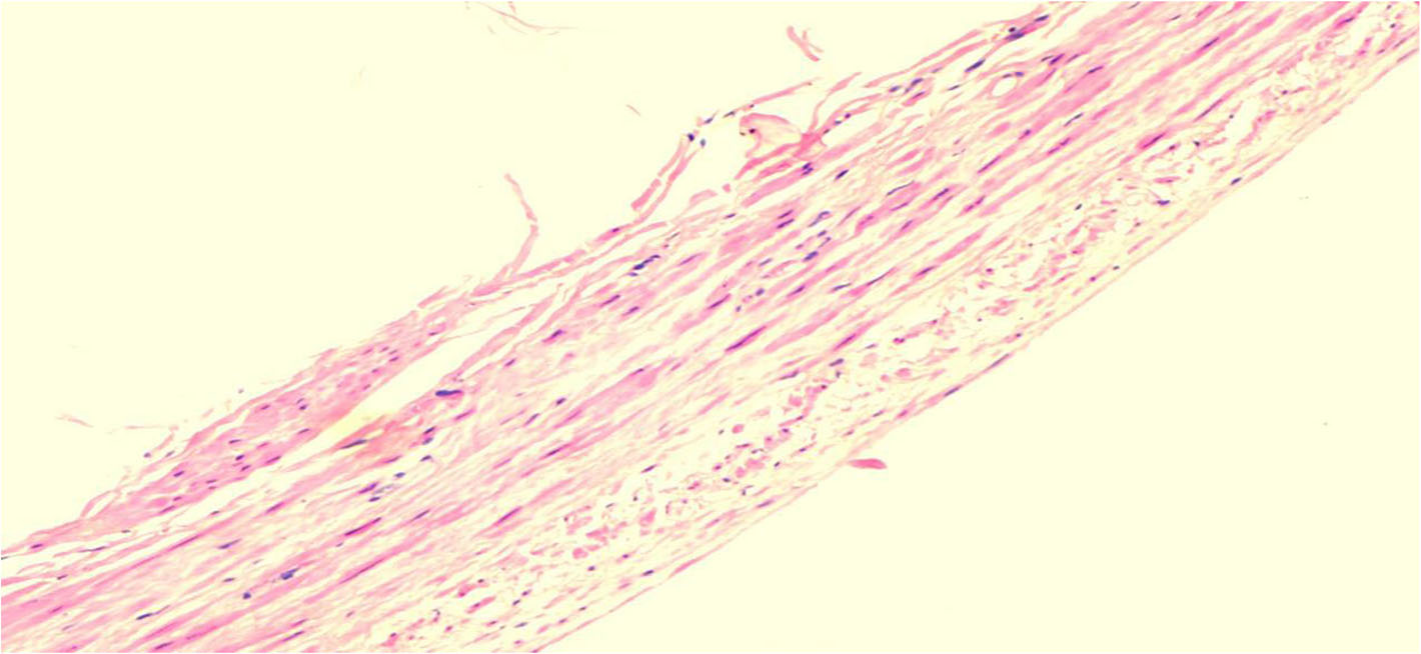
This is the wall tissue of a venous aneurysms stained with hematoxylin-eosin staining

The mediastinal drainage tube was removed on the third day after the operation, and the patient was discharged from the hospital 2 days later. Postoperative pathology showed that the specimen was thinned venous wall tissue (Fig. 3). He was followed up for 6 months, and there was no obvious abnormality in the chest enhanced CT, with good recovery.

## Discussion

Brachiocephalic venous aneurysms often display no specific clinical symptoms. They are often found by further examinations after physical examination of abnormal mediastinum [5]. In this case, the patient had an irritating cough due to the large aneurysm and compression. The chest CT scan only detected abnormal mediastinal masses, which requires enhanced CT or MRI to identify. When thrombosis occurs, it is manifested as a localized filling defect in the lumen. If blind biopsy is performed, serious or even fatal complications may occur [6]. MRI of the chest shows abnormal blood flow at the base of the venous aneurysm, which shows better imaging than CT.

The reports of brachiocephalic vein aneurysms are often based on individual cases, lacking of unified treatment guidelines. Conservative treatment includes: for fusiform and small cystic brachiocephalic vein aneurysms, which rarely cause aneurysm enlargement, compression, rupture, etc., therefore, long-term antiplatelet conservative treatment and regular review are sufficient if there is no significant change in the size and shape of the aneurysm [7]. But if the symptoms are aggravated, the aneurysm is enlarged, or imaging suggests that related complications occur, surgical intervention should be applied.

Scholars are constantly exploring that endovascular treatment is less traumatic for patients, and recovery is quicker after surgery. Gaopo Cai [8] reported that the use of an endovascular stent to treat a case of left brachiocephalic vein aneurysm have achieved satisfactory early results, but during the 12–18 months follow-up stent thrombosis gradually appeared, and its long-term effect needs further checked. In 2014, Jargiello [9] reported percutaneous transcatheter thrombin injection for the treatment of cystic venous aneurysms. After intraoperative injection of thrombin, balloon expansion is used to seal the entrance of the aneurysm to achieve the therapeutic effect. It is suitable for patients with narrow and long aneurysm necks, or small aneurysms, but their allergic reactions and intraoperative and postoperative embolization complications need to be vigilant. For this case, because of our insufficient experience in endovascular treatment and the patient was unwilling to try this new technique, surgery was therefore used.

We believe that although the surgical path of median thoracotomy is more traumatic, it gives the surgeon a full view during the operation and facilitates the observation of the anatomical relationship between the venous aneurysm and the surrounding tissues, and can provide help for the extracorporeal circulation surgery when necessary. This method is safe and effective. After blocking the blood flow on both sides of the aneurysm, we clamped the neck of the aneurysm, excised the aneurysm, and did double-layer continuous suture with 5–0 prolene thread for angioplasty. During the process, we paid attention to the diameter of the venous lumen, to prevent excessive sutures from causing stenosis. The results of the postoperative follow-up visit were satisfactory. The patient’s symptoms were significantly improved after the operation. Cheng Fang [10] also reported a case of left brachiocephalic vein aneurysm with accumulated superior vena cava. They used autologous pericardium tissue from the patient to reconstruct venous blood vessels, and also achieved a good therapeutic effect. In addition, autologous pericardium has the advantages of strong plasticity, easy access, and better immunocompatibility [11], so that patients do not need anticoagulant treatment after surgery. This method is beneficial when the aneurysm is large and other blood vessels are dilated.

## Conclusion

In summary, once the space-occupying lesions in the mediastinum were found and the diagnosis of venous aneurysms is not ruled out, it is necessary to further examine the chest with enhanced CT or MRI. The treatment methods are diverse, and the clinical needs should be based on the type, size, location, scope of the venous aneurysm, and complications. Appropriate method should be chosen. For larger cystic brachiocephalic venous aneurysms, it is feasible to perform surgical resection and angioplasty to reconstruct the venous lumen.

## Data Availability

Not applicable.

## Abbreviations

CT: Computed topography
MRI: Magnetic resonance imaging
